# Prophylaxis for iron deficiency anemia using ferrous sulfate among infants followed up at a primary healthcare unit in the municipality of Embu-SP (2003/2004)

**DOI:** 10.1590/S1516-31802008000200006

**Published:** 2008-03-06

**Authors:** André Fernando Shibukawa, Edina Mariko Koga da Silva, Wilson André Ichiki, Maria Wany Louzada Strufaldi, Rosana Fiorini Puccini

**Keywords:** Anemia, iron-deficiency, Ferrous sulfate, Child care, Disease prevention, Primary health care, Anemia ferropriva, Sulfato ferroso, Cuidado da criança, Prevenção de doenças, Atenção primária

## Abstract

**CONTEXT AND OBJECTIVE:**

Iron deficiency anemia is an important public health problem in Brazil. In the municipality of Embu, a population study in 1996 found anemia prevalence of 68.5% among children aged one to two years. From these data, prescription of prophylactic ferrous sulfate was instituted in 1998 for children under two years old followed up within the children's healthcare program. After five years of intervention, the prevalence of anemia and associated factors were investigated among children aged 12 to 18 months to whom guidance for prophylactic ferrous sulfate use had been given.

**DESIGN AND SETTING:**

Cross-sectional study covering October 2003 to June 2004 at a primary healthcare unit in Embu.

**METHODS:**

A randomized sample of children aged 12 to 18 months to whom guidance for prophylactic ferrous sulfate use had been given was obtained. Hemoglobin was measured in capillary blood, using HemoCue^®^ apparatus. Hemoglobin < 11 g/100 dl was taken to indicate anemia.

**RESULTS:**

The sample comprised 118 children and anemia was found in 41.5%. There was no statistically significant association between anemia presence and the variables of sex, birth weight, neonatal intercurrences, chronic diseases, breastfeeding or iron supplementation use. There was a statistically significant association (p = 0.03) between anemia presence and per capita income, such that the higher the income was, the lower the prevalence of anemia was.

**CONCLUSION:**

The prophylaxis program against iron deficiency anemia did not achieve the expected results. New strategies must be considered in the light of the magnitude of the problem.

## RESUMO

**CONTEXTO E OBJETIVO:**

A anemia ferropriva é um importante problema de saúde pública no Brasil. No município de Embu, estudo populacional realizado em 1996 verificou prevalência de anemia de 68,5% em crianças de um a dois anos. A partir desses resultados, o uso de sulfato ferroso profilático foi introduzido em 1998 no Programa de Saúde da Criança para as crianças a partir do desmame até os dois anos de idade. Após cinco anos de intervenção, avaliou-se a prevalência de anemia e os fatores associados, em crianças de 12 a 18 meses de idade que receberam orientação para uso profilático de sulfato ferroso.

**TIPO DE ESTUDO E LOCAL:**

Estudo transversal analítico realizado no período de 10/2003 a 06/2004 em Unidade Básica de Saúde, Embu.

**MÉTODO:**

Amostra aleatória de crianças entre 12 a 18 meses que receberam orientação do uso de ferro profilático. Mensuração de hemoglobina em sangue capilar através do aparelho portátil HemoCue^®^. Hemoglobina < 11 g/100 dl foi considerada anemia.

**RESULTADOS:**

A amostra foi composta por 118 crianças. A prevalência de anemia foi de 41,5%. Não foi observada associação estatisticamente significativa entre a anemia e as variáveis: sexo, peso ao nascer, intercorrências neonatais, doenças crônicas, internação anterior, aleitamento materno, uso de suplementação de ferro, estado nutricional. Verificou-se associação estatisticamente significante (p = 0,03) entre a presença de anemia e renda *per capita*, em que a maior renda associou-se a menor prevalência de anemia.

**CONCLUSÃO:**

O programa de profilaxia da anemia ferropriva não alcançou os resultados esperados. Novas estratégias devem ser consideradas frente à magnitude do problema.

## INTRODUCTION

Anemia is defined as a pathological process in which the hemoglobin concentration is abnormally low, with due regard for variation according to age, sex and altitude above sea level. For children aged between six months and six years, the World Health Organization (WHO) has proposed a figure of 11 g/dl as the lower limit of normality.^1^ Nutritional anemia is a consequence of lack of one or more of the following nutrients (whatever their origin is): iron, folic acid, vitamin B_12_, copper and vitamins A, C and E. Iron deficiency alone is the most frequent of these shortages and reaches around 25% of the world population.^2^

The distribution of iron deficiency anemia is universal, but the prevalence in developing countries is four times greater than what is found in developed countries.^2^ De Mayer et al. (1985) produced a compilation of studies on anemia for WHO and observed that, in less-developed regions, it affected between 2% and 97% of children aged less than five years. On the other hand, in developed countries, these authors found prevalence between 0% and 34%. Although low income, as a conditioning factor for insufficient food consumption, plays an important role in the occurrence of iron deficiency anemia, other factors associated with this also act as determining factors, including low birth weight, precarious access to health services, greater incidence of infectious processes, and inadequate education and sanitation.^3,4^

In Brazil, most studies on the prevalence of anemia have been conducted among restricted population groups or among groups coming from health clinics. Although these studies were carried out in different regions, with different methodologies, they have shown a tendency towards increasing prevalence of anemia. This was observed by Monteiro et al.^5^ in the municipality of São Paulo, in population-based studies carried out in 1984-5 and 1995-6.

In the light of this situation of extreme seriousness, interventions have been proposed. It is recognized that iron deficiency anemia prevention can be based on four types of approach: nutritional education and improvement of the quality of the diet offered, including encouragement of exclusive breastfeeding; iron supplementation through medication; infection control; and fortification of basic foods with iron.^4^ Iron supplementation through medication has the advantage of producing rapid changes in nutritional iron status. Moreover, it is a specific strategy that can be directed to population groups with high iron needs and therefore at greater risk of presenting lack of this mineral. Since 1995, the Brazilian Society of Pediatrics has been recommending iron supplementation through medication for all children, with specific dosages for low birth weight infants, premature infants and full-term infants (Brazilian Society of Pediatrics Committee, 1995). On the other hand, this approach presents difficulties regarding adherence and correct administration, in that a variety of factors can interfere in the absorption of such medications.

In the municipality of Embu (State of São Paulo) up to 1998, in accordance with regulations established by the Municipal Health Department, prophylactic prescription of iron medications was restricted to children with low birth weight or premature infants. Children born at full term, and even those at the weaning stage, did not receive such guidance systematically because of insufficiency of the medication in the municipality's primary care network. At that time, priority was given to the distribution of medications for treating children who already presented anemia. In a study conducted in 1996, anemia prevalence of 40.7% was found among children aged under five, and 68.5% among children between one and two years old.^6^ From these data, prescription of prophylactic ferrous sulfate was instituted throughout the healthcare network in 1998, at a dose of 1 to 2 mg/kg per day from the time of weaning until two years of age, for children followed up within the children's full healthcare program, as an addition to the previous regulations relating to children of low birth weight and premature infants. The healthcare units then started to make this medication available, with the aim of complying with the regulations established.

Studies that assess the impact of interventions have fundamental importance. The specific case of prophylactic medication for iron deficiency anemia involves difficulties relating to adherence to prolonged use of the medication and its correct administration, and also irregularity in its distribution. Such studies should be performed in this case with the objectives of reinforcing the maintenance of adequate measures and redirecting actions when they are shown to be ineffective.

## OBJECTIVE

Following five years of intervention (prophylactic medication for iron deficiency anemia), it was considered necessary to investigate the prevalence of anemia among children aged 12 to 18 months who were being followed up within the children's full healthcare program at the “Maria Marluce Gouveia” Primary Healthcare Unit in the Jardim Santo Eduardo district and had received guidance for prophylactic use of ferrous sulfate, and to study the factors associated with anemia in this group of children.

## METHODS

*Type of study*: This was a cross-sectional study. *Location*: The study was conducted in a primary healthcare unit in Embu, a municipality in the Metropolitan Region of São Paulo. In 2003, the year when this study was conducted, the population of Embu was estimated to be 225,629 inhabitants. More than 95% of the homes had treated water and public garbage collection available, and 57% were connected to the sewage network. The infant mortality rate for 2003 was 13.98 per thousand live births, and perinatal conditions predominated.^7^ The municipal healthcare network in 2003 comprised nine primary healthcare units, two emergency services, one psychosocial care center, one occupational healthcare center and one referral center for adolescent care. The Pirajussara Hospital, located at the boundary between the municipalities of Embu and Taboão da Serra and administrated by the State Health Department, has 240 beds and has constituted a secondary referral center for the municipality since 1999.

*Population*: The study population consisted of a randomized sample of children aged 12 to 18 months who were being followed up within the children's full healthcare program and for whom prophylactic ferrous sulfate had been prescribed upon weaning. This information was obtained from the patients’ records for the period from October 2003 to June 2004.

*Sample*: The sample size calculation was performed using the Epi-Info program, version 6.01 (Centers for Disease Control, CDC, and WHO, 1994). This calculation assumed that the proportion of the population expected to present anemia was 70.0% (range: 60% to 80%), in conformity with a previous study.^4,5^ This percentage was then applied to the estimated population of children in the municipality within this age group (year 2000), with a 95% confidence interval, to give a calculated sample size of 78 children.

*Data collection*: After analyzing the patients’ records, the children who fulfilled the inclusion criteria were invited, by means of a letter, to attend the unit at an arranged time and date. On this day, the following procedures were carried out:

- Hemoglobin measurement in capillary blood, by means of the portable HemoCue^®^ apparatus (HemoCue AB, Ängelholm, Sweden). Children whose hemoglobin was lower than 11 g/100 dl were considered to be anemic.^1^
- Interview with the adults responsible for the child, to obtain information on the following variables relating to the child: birth weight, neonatal intercurrences, chronic diseases, previous hospitalization, breastfeeding and use of iron supplementation; and on the following variables relating to the family: mother and father's ages, mother and father's schooling, mother and father's present employment situation, and per capita income.
- Anthropometry:
  – Weight: The children's weights were measured using a basket-type Filizola^®^ balance, with a capacity of up to 16 kg and a scale graduated in ten-gram divisions.
  – Height: A horizontal-type wooden anthropometer was utilized for measuring the children's height, with a scale graduated in millimeters. The children were measured in dorsal decubitus, with the head fixed, legs extended and feet at ninety degrees.

For the nutritional evaluation, anthropometric indices were calculated: weight for age (W/A), height for age (H/A) and weight for height (W/H). The reference taken was the curves from the National Center for Health Statistics (NCHS, 1977). By using the distribution of the indices expressed as z-scores, children were considered to be malnourished if at least one of their anthropometric indices was more than two standard deviations below the reference.^8^

*Data analysis*: The text processing, data entry and data analysis were done on an IBM-compatible computer, using the Epi-Info 6 microcomputer system program, version 6.01.

*Statistical analysis*: To compare the categorized variables between the two groups, the chi-squared test was utilized, as calculated by the Epitable program of Epi-Info 6.01. A logistic regression model was used for multivariate analysis, with anemia as the response variable, by means of the multiple logistic regression model (MULTLR program).^9^ For all the statistical tests, the significance level of 5% was adopted (α = 0.05). When the calculated p-value (minimum significance level) allowed rejection of the nullity hypothesis, it was flagged with an asterisk (p < 0.05). To include variables in the logistic regression model, a significance level of 10% (0.10) was taken.

All the children for whom a diagnosis of anemia was established were given appointments at the healthcare unit for additional diagnostic tests and follow-up.

This project was approved by the Research Ethics Committee of Universidade Federal de São Paulo (Unifesp), and the persons responsible for the children signed written declarations of informed consent, upon inclusion in the study.

## RESULTS

The sample comprised 118 children with a mean age of 15.8 months (standard deviation, SD = 1.8 months), of whom 58 (49.2%) were male. Anemia was found to be present in 49 children (41.5%).

With regard to the type and number of visits to the primary healthcare unit, there was a mean of 4.6 (SD = 2.1) child-rearing consultations, 1.2 (SD = 0.9) nursing consultations and 0.9 (SD = 1.0) other consultations.

Prophylaxis using ferrous sulfate was started when the children were at a mean age of 9.8 months (SD = 3.4) and, although 65.3% of them were reported to be still using the medication, the administration was inadequate for 19.5% of the children (underdosage).

Among all the children analyzed, 11.1% had had low birth weight, 6.8% had had neonatal intercurrences and most had been premature. There were 2.5% with chronic diseases (wheezing attacks, convulsions and tracheal stenosis) and 8.2% had previously been hospitalized, mainly for pulmonary diseases and diarrhea. With regard to breastfeeding, 90.7% of the children had been started on breastfeeding, 64.4% had predominantly been breastfed for four months or more, and 33.1% were still being breastfed (Table 1).

**Table 1.** Frequency of anemia and associations between anemia and the variables relating to the children. Embu, 2003-2004

Variables	Without anemia		With anemia		Total	p
	n	%	n	%	n	
**Sex**						
Male	31	53.4	27	46.6	58	0.3
Female	38	63.3	22	36.7	60	
**Birth weight (g)**						
< 2500	9	69.2	4	30.8	13	0.7
2500 – 3000	16	57.1	12	42.9	28	
≥ 3000	44	57.1	33	42.9	77	
**Neonatal intercurrences**						
Yes	3	37.5	5	62.5	8	0.3
No	66	60.0	44	40.0	110	
**Chronic diseases**						
Yes	3	100.0	0	0.0	3	0.3
No	66	57.4	49	42.6	115	
**Previous hospitalization**						
Yes	11	78.6	3	21.4	14	0.1
No	58	55.8	46	44.2	104	
**Started breastfeeding**						
Yes	64	59.8	43	40.2	107	0.5
No	5	45.5	6	54.5	11	
**Predominantly breastfed ≥ 4 months**						
Yes	47	61.8	29	38.2	76	0.3
No	22	52.4	20	47.6	42	
**Still being breastfed**						
Yes	26	66.7	13	33.3	39	0.3
No	43	54.4	36	45.6	79	
**Using iron supplementation**						
Yes	45	58.4	32	41.6	77	0.5
No	24	60.0	16	40.0	40	
Without information	0	0.0	1	100.0	1	
**Nutritional evaluation (Z-score)**						
Eutrophic	62	56.4	48	43.6	110	0.1
Malnourished	7	87.5	1	12.5	8	

The parents’ characteristics and per capita income of the sample studied are presented in Table 2. It was observed that the mothers and fathers were generally 20 years old or over and had a low level of schooling. Among the fathers, 80.2% were employed, while 27.4% of the mothers were employed. With regard to the families’ socioeconomic level, 46.8% had a per capita family income of less than 0.5 minimum salaries per month.

**Table 2.** Frequency of anemia and relationship between anemia and the variables relating to sociodemographic factors. Embu, 2003-2004

Variables	Without anemia		With anemia		Total	p
	n	%	n	%	n	
**Mother's age**						
< 20 years old	9	90.0	1	10.0	10	0.05
20 – 30	38	51.4	36	48.6	74	
≥ 30	22	64.7	12	35.3	34	
**Mother's education**						
Primary school incomplete	28	60.9	18	39.1	46	0.20
Primary school complete	27	43.5	15	56.5	42	
Secondary school complete	10	43.5	13	56.5	23	
High school and more	3	50.0	3	50.0	6	
Without information	0	0.0	1	100.0	1	
**Mother employed**						
Yes	23	71.9	9	28.1	32	0.06
No	45	52.9	40	47.1	85	
Without information	1	100.0	0	0.0	1	
**Father's age**						
< 20 years old	8	72.7	3	27.3	11	0.10
20 – 30	29	49.2	30	50.8	59	
≥ 30	32	66.7	16	33.3	48	
**Father's education**						
Primary school incomplete	31	58.5	22	41.5	53	0.30
Primary school complete	12	44.4	15	55.6	27	
Secondary school complete	7	53.8	6	46.2	13	
High school and more	1	100.0	0	0.0	1	
**Father employed**						
Yes	52	61.2	33	38.8	85	0.30
No	10	47.6	11	52.4	21	
Without information	7	58.3	5	41.6	12	
**Per capita income**						
< 0.5 minimum salaries	24	46.2	28	53.8	52	0.03
0.5 – 1 minimum salaries	28	66.7	14	33.3	42	
≥ 1 minimum salaries	13	76.5	4	23.5	17	

The analyses of the associations between anemia and the variables relating to the children and their families are presented in Tables 1 and 2, respectively. There was a statistically significant association (p = 0.03) between the presence of anemia and per capita income, such that the higher the income was, the lower the prevalence of anemia was. In the multivariate analysis, only the income remained statistically significant. For families with a per capita income of less than 0.5 minimum salaries, the chance that the child would present anemia was almost three times greater than among the higher-income families (odds ratio, OR = 2.7; 95% confidence interval, CI: 1.2 – 5.7).

## DISCUSSION

Some limitations of the present study must be noted. The sampling was restricted to children enrolled at primary healthcare units and therefore does not represent the population. The present analysis was capable of indicating the children with anemia, but without identifying some of the important factors that are associated with anemia, such as their present diet. Moreover, no measurement was made of the low adherence to supplementation, which is a prescription for chronic use. Some authors have also questioned the use of hemoglobin assaying as a means of screening for iron deficiency anemia, because this is a test with low sensitivity and low specificity that may fail to diagnose or may underdiagnose iron deficiencies in children who present normal hemoglobin levels.^10,11^ It is however accepted that the endemic occurrence of this condition during infancy fundamentally results from a lack of this mineral.^12^ It must also be considered that capillary assaying, as was done in our study, presents lower levels than the concentrations found via venous puncture,^13^ although this method is widely utilized in population-based studies because of its practicality.

Another question that has been greatly discussed concerns hemoglobin concentration levels. Studies in which the hemoglobin and ferritin levels were simultaneously analyzed in children^14,15^ have concluded that in infants the cutoff point for identifying anemic children is at around 9.7 g/dl for hemoglobin. WHO recently set up a working group with the purpose of reviewing the hemoglobin levels that are currently established.^16^

In a study conducted in the municipality of Embu in 1996, among a population-based sample of children aged under five years, no statistically significant difference in anemia prevalence was found for children aged under 15 months (the routine age group for the children's full healthcare program), between those followed up within the program (72.0%) and those who were not (76.0%).^17^ In 2003, for the present study, only the children followed up within the program for whom ferrous sulfate had been prescribed were taken into consideration. Although a decrease in anemia prevalence among children aged 12 to 18 months in relation to 1996 was observed, this rate remained very high (41.5%). These findings are similar to those from other Brazilian studies, such as the studies by Levy-Costa and Monteiro in the municipality of São Paulo (45.2%),^18^ Silva et al. in the municipality of Porto Alegre, State of Rio Grande do Sul (47.8%),^19^ or Almeida et al. in Pontal, State of São Paulo (62.5%),^20^ which found that the prevalence could even be greater at this same age group. These findings show that anemia remains a serious public health problem and that prophylaxis alone does not achieve the results expected.

With regard to the factors associated with anemia, it was found in the present study that some of them that are already well known in the literature as risk or protective factors did not present any statistically significant association, possibly because of the sample size. These factors were the presence of neonatal intercurrences, breastfeeding and the father and mother's employment situations. The employment situations were certainly related to income and were subsequently confirmed as having an association with the presence of anemia.

Nevertheless, limitations on consolidating the relationships with associated factors also appear in other studies. A study by Santos et al.^21^ analyzed the prevalence of anemia among children aged under six years in the municipality of Pelotas (State of Rio Grande do Sul) and found that the risk factors for anemia were younger age for the child, nonwhite color, large-sized family and social class E (in comparison with D). On the other hand, the availability of piped water inside the home and greater birth weight were shown to be protective factors. In another study that assessed the anemia prevalence (47%) among children enrolled in daycare centers in Rio de Janeiro, Matta et al.^22^ found that the children with lower weight for age and height for age who were under two years of age and living in homes with large numbers of people, and whose parents had low levels of schooling, were the ones who were most vulnerable to anemia. In a study by Silva et al.,^19^ the determinants for anemia were age group between 12 and 23 months, per capita income less than one minimum salary and the presence of two or more siblings aged under five years.

Many studies have made an association between low birth weight and the occurrence of anemia.^5,21,23,24^ This association can be explained as a consequence of reduction in the reserves accumulated during gestation, along with the interaction with other factors of socioenvironmental nature: infections, insufficient and inadequate diet, and difficulties in obtaining access to health services.

Greater prevalence of anemia in association with malnutrition has been found in some studies,^24^ although anemia often occurs independently of malnutrition. Today, the increased prevalence of anemia is accompanied by lower rates of malnutrition.^25^

This can be explained by the greater iron needs presented by eutrophic children. A similar situation was also observed by Santos et al.^21^ in a population in which 10% of the children aged under six years were more than two standard deviations below the reference scores for weight/age and height/age, and there was no association between anemia and the anthropometric indicators. These authors explained that this was possibly due to low iron levels plus low bioavailability of this mineral in the habitual diet of the children studied, regardless of the weight and height that had been attained. In the present study, two children who were malnourished did not present anemia.

Per capita income constitutes an important determining factor for anemia, such that children from families with lower income present greater prevalence than do others with greater income.^20,25^ A study conducted in Belém (State of Pará) among infants attended at a school health center demonstrated an association between iron deficiency and per capita income of less than or equal to one minimum salary.^26^ In the present study too, an association was seen between income and anemia.

Fortification of basic foods has been shown to be effective in reducing the prevalence of iron deficiency anemia, and this is a well-established practice in several countries. However, even though iron fortification may have a role in preventing iron deficiency, and even if a population has access to iron-enriched foods, some groups are more vulnerable and require oral supplementation to prevent and control iron deficiency at certain stages of life. In places where iron fortification is viable, this intervention may serve as an entry point for a mixed strategy, since fortification may reduce the iron deficiency in all sectors of the population and may make other interventions more effective.^25^ Kapur et al.^27^ suggested that dietary diversification by means of nutritional education and food fortification could reduce the prevalence of moderate to serious anemia among children. On the other hand, a study by Getman et al.^28^ did not find any decrease in the prevalence of iron deficiency or anemia among children who received multivitamins with or without prophylactic doses of iron between the ages of six and nine months.

Torres et al.^24^ observed an improvement in hemoglobin levels among children who were receiving prophylactic ferrous sulfate at a primary healthcare unit in the municipality of São Paulo, although there was a large sample loss (around 50%) and also low adherence to treatment (only 52.5% were correctly administering the medication).

There are a variety of factors relating to the success of iron supplementation programs. Among these, the effectiveness of supplementation depends on widespread distribution and access to the supplement. Another factor involved relates to organization, training and education, along with the development of communication strategies. Other important factors include the degree of participation by the community and the links to other health and nutrition programs, including other interventions relating to iron as fortification for foods.^25^

## CONCLUSIONS

In the present study, the expected result of possibly decreased anemia prevalence following the preventive measure of introducing ferrous sulfate for children at the time of weaning was not achieved. The hypotheses for this, which will need to be proven in future studies, should take into account the possible low adherence to treatment, which encompasses several angles, going from correct guidance for and understanding by families regarding the need to use the medication, to limitations caused by chronic use of medications, considering that the medication must be offered for a prolonged period and also has a disagreeable taste.

In view of the high anemia rate observed (41.5%), the existing preventive programs need to be adapted so as to incorporate new strategies, such as prophylaxis using medication, encouragement of breastfeeding and dietary education.
